# Genomics and bacterial pathogenesis.

**DOI:** 10.3201/eid0605.000509

**Published:** 2000

**Authors:** G M Weinstock

**Affiliations:** University of Texas, Houston Medical School, Houston, Texas 77030, USA.

## Abstract

Whole-genome sequencing is transforming the study of pathogenic bacteria. Searches for single virulence genes can now be performed on a genomewide scale by a variety of computer and genetic techniques. These techniques are discussed to provide a perspective on the developing field of genomics.


### Genomics and Bacterial Pathogenesis

**George M. Weinstock**
University of Texas, Houston Medical School, Houston, Texas, USA

Whole-genome sequencing is transforming the study of pathogenic bacteria. Searches for single virulence genes can now be performed on a genomewide scale by a variety of computer and genetic techniques. These techniques are discussed to provide a perspective on the developing field of genomics.

Twenty-five years ago, the development of molecular biology and recombinant DNA technology promised breakthroughs in infectious disease research. Since then, these methods have slowly teased out molecular secrets of microbial infection, gene by gene. Now, with the advent of whole-genome sequencing, a new revolution in infectious disease research has begun. Genomics is a top-down approach to the study of genes and their functions, taking advantage of DNA sequences of complete genomes. Determining the DNA sequence of a complete genome is a major activity of genomics. Although basic DNA-sequencing methods have remained the same, advances in automation and informatics enable determination of whole microbial genome sequences in <2 years. Complete knowledge of an organism's genetic makeup allows exhaustive identification of candidates for virulence genes, vaccine and antimicrobial targets, and diagnostics. The genomes of at least 13 pathogenic bacteria have been sequenced (Table 1), representing >20,000 putative genes. The genomes of at least 28 other pathogenic bacteria are being sequenced, promising >40,000 additional genes. This tally does not include an equally large number of nonpathogenic bacteria undergoing whole-genome sequence analysis. These new data dwarf previous methods of gene discovery, allowing many new genetic approaches to understanding pathogenesis.

### Raw Material

Genome projects produce different types of data, depending on the stage and goals of the project (Table 2). The goal of most projects is a

Address for correspondence: George M. Weinstock, Department of Microbiology and Molecular Genetics, University of Texas, Houston Medical School, 6431 Fannin, Houston, TX 77030, USA; fax: 713-500-5499; e-mail: georgew@utmmg.med.uth.tmc.edu.

Table 1. Whole-genome sequencing of bacterial pathogens[a]

| Bacterium | Status (ref.) |
|---|---|
| *Actinobacillus actinomycetemcomitans* | In progress |
| *Bacillus anthracis* | In progress |
| *Bartonella henselae* | In progress |
| *Bordetella bronchiseptica* | In progress |
| *B. parapertussis* | In progress |
| *B. pertussis* | In progress |
| *Borrelia burgdorferi* | Finished (1) |
| *Campylobacter jejuni* | Finished |
| *Chlamydia pneumoniae* | Finished (2) |
| *C. trachomatis* | Finished (3) |
| *Clostridium difficile* | In progress |
| *Enterococcus faecalis* | In progress |
| *Escherichia coli* K12 | Finished (4) |
| *E. coli* O157:H7 | In progress |
| *Haemophilus influenzae* | Finished (5) |
| *Helicobacter pylori* | Finished (6,7) |
| *Listeria monocytogenes* | In progress |
| *Mycobacterium avium* | In progress |
| *M. leprae* | In progress |
| *M. tuberculosis* | Finished (8) |
| *Mycoplasma genitalium* | Finished (9) |
| *M. mycoides* | In progress |
| *M. pneumoniae* | Finished (10) |
| *Neisseria gonorrhoeae* | In progress |
| *N. meningitidis* | In progress |
| *Porphyromonas gingivalis* | In progress |
| *Pseudomonas aeruginosa* | In progress |
| *P. putida* | In progress |
| *Rickettsia prowazekii* | Finished (11) |
| *Salmonella* serotype Typhi | In progress |
| *S.* Typhimurium | In progress |
| *Shigella flexneri* | In progress |
| *Staphylococcus aureus* | In progress |
| *Streptococcus mutans* | In progress |
| *S. pneumoniae* | In progress |
| *S. pyogenes* | In progress |
| *Treponema denticola* | In progress |
| *T. pallidum* | Finished (12) |
| *Ureaplasma urealyticum* | Finished |
| *Vibrio cholerae* | In progress |
| *Yersinia pestis* | In progress |

[a]Much of these data were taken from the TIGR website (see Table 2). In-progress genome projects are those that are funded but not yet complete.

Table 2. Availability of sequence data

| Internet site | Organization | Description |
|---|---|---|
| www.ncbi.nlm.nih.gov/Entrez/Genome/org.html | National Institute of Biotechnology Information | Many genomes represented |
| http://www.tigr.org/tdb/index.shtml | The Institute for Genomic Research | Genomes sequenced by TIGR |
| www.stdgen.lanl.gov | Los Alamos National Laboratory | Sexually transmitted disease pathogens |
| http://www.micro-gen.ouhsc.edu/ | University of Oklahoma | Genomes sequenced at the Univ. Oklahoma |
| www.pasteur.fr/recherche/banques/Colibri/ | Institut Pasteur | Colibri, database of the *Escherichia coli* genome |
| pedant.mips.biochem.mpg.de/ | Pedant | Many genomes represented |
| http://www.ncgr.org/research/sequence/ | National Center for Genome Resources | Many genomes represented |
| http://www.kazusa.or.jp/cyano/ | Kazusa DNA Research Institute | Cyanobase, cyanobacterial genome information |
| www.sanger.ac.uk/Projects/Microbes/ | Sanger Centre | Genomes sequenced by the Sanger Centre |
| www.genetics.wisc.edu/ | University of Wisconsin | Genomes sequenced by the Univ. Wisconsin Genome Center |
| www.zmbh.uni-heidelberg.de/M_pneumoniae/genome/Results.html | University of Heidelberg | *Mycoplasma pneumoniae* genome |
| utmmg.med.uth.tmc.edu/treponema/tpall.html | Univ. Texas Houston Medical School | *Treponema pallidum* |
| http://chlamydia-www.berkeley.edu:4231/ | Univ. Cal. – Berkeley | *Chlamydia* genomes |
| evolution.bmc.uu.se/~siv/gnomics/ | Uppsala University | *Rickettsia prowazekii* |
| http://www.genomecorp.com/sequence_center/index.html | Genome Therapeutics Corp. | Genomes sequenced at GTC |
| genome.wustl.edu/gsc/Projects/bacteria.shtml | Washington University | Genomes sequenced at Washington Univ. |
| http://www.genoscope.cns.fr/externe/English/Projets/Resultats/rapport.html | Genoscope | Genomes sequenced at Genoscope |
| www.genome.washington.edu | Univ. of Washington | *Pseudomonas aeruginosa* |
| www.pseudomonas.com | Pathogenesis Corp. | *P. aeruginosa* |
| pbil.univ-lyon1.fr/emglib/emglib.html | Enhanced Microbial Genomes Library | Many genomes represented |
| www.pasteur.fr/recherche/banques/TubercuList/ | Institut Pasteur | TubercuList, database of the *Mycobacterium tuberculosis* genome |

finished contiguous DNA sequence of the bacterium's chromosome(s). The error frequency in a finished sequence has never been precisely measured but is thought to be one error (frameshift or base substitution) in $10^3$ to $10^5$ bases. Other types of errors, such as rearrangements, are probably even more rare. Even at the higher end of this error frequency, approximately one error per gene, the sequence is still very useful for database searches and most applications.

Finished genome sequences are annotated to varying degrees. The two most important annotations are the predicted protein coding sequences, generally called open reading frames (ORFs), and what they resemble in database searches (see below). Strictly speaking, an ORF is any stretch of codons that does not include a chain termination codon; however, only a subset of all the ORFs present in the genomic sequence actually encodes proteins and is used in genome annotation. These ORFs are identified by predicting coding sequences. The predictions are 90% to 95% accurate. In addition, many untranslated RNAs (mainly tRNA and rRNA genes) are identified and annotated. Various other features may be part of the annotation, including elements of the predicted protein structure, such as secondary structure motifs and membrane spanning regions. Unfortunately, annotation rarely extends to noncoding regions, where promoters and regulatory signals reside. Similarly, structural features of DNA (e.g., Z-DNA) are rarely analyzed, which may bear on regulation or genome structure. At this time, the emphasis is overwhelmingly on gene products since these convert sequence data into useful products.

A near-universal trend among public (but not private) genome projects is the early release of unfinished sequence data, sometimes referred to as (rough) draft sequences. This release can occur when as little as 1x coverage (coverage being the number of bases read in DNA sequencing reactions, divided by the genome size) of the genome has been obtained by random sequencing; for an average-size 2-MB genome, this may mean 4,000 sequencing reads. Most genomes will have been sequenced at least once, although the sequence will have a high error rate and many gaps, and some regions of the genome will not be represented. These random sequence reads are assembled by a computer program that looks for overlaps between the individual sequences and generates consensus sequences, i.e, a sequence in agreement with most of the individual reads (present in stretches of contiguous nucleotides or contigs). Since there are many gaps in the sequence, hundreds to thousands of contigs are produced by this process, with a wide range of sizes (typically from 100s to 10,000s of bases)—although always much smaller than the total genome. Collections of contigs can be searched for matches to sequences of interest, allowing identification of relevant contigs and specific DNA sequences within them. This analysis prior to release of the completed sequence speeds the application of results from genome projects.

### Finding Hints in Sequences

Several approaches can be used to analyze whole-genome sequences for candidate virulence factors and for vaccine and antimicrobial targets. Comparing predicted coding sequences to sequences in databases (e.g., GenBank), using the BLAST program (13,14) identifies matches to known genes. Typically, approximately 20% of the predicted ORFs in a genome do not match anything in GenBank, while another 10%–20% match genes of unknown function, often discovered in other genome projects. The fraction of genes of unknown function in a genome has been remarkably constant in microbial genome sequences, regardless of the number of genomes sequenced and available for comparison. Thus, the comparison approach is useful in recognizing good candidates among genes whose functions have been described; it is not particularly useful in discovering new virulence functions or motifs.

For microbes related to well-studied pathogens, such as gram-positive cocci or gram-negative enteric pathogens, comparing sequence data yields many database matches or "hits." For organisms more distantly related to well-studied groups, results are more modest. When this approach was used for the spirochete *Treponema pallidum*, only 70 genes out of 1,041 could be recognized as potential virulence factors (15). Since a number of these had previously been described as antigens or membrane proteins without a function implicating them in infection, only half of the 70 genes could be matched to a function associated with virulence or host interaction in another pathogen. Of these, the evidence for some of the existing database annotations was slim, at times only theoretical and not based on solid experiments. These spurious annotations can be readily perpetuated because of the volume of new genes entered without critical evaluation. Thus for *T. pallidum*, for which approximately 40% of the total ORFs did not match a gene with any annotated function (12), virulence factors are likely to be novel, and other methods for their discovery are needed.

Databases that do not search for matches to whole genes or proteins can also be searched. These include databases of protein motifs such as BLOCKS (a database of conserved regions of protein families, obtained from multiply aligned sequences [16,17]) and ProDom (18,19). Hits to these databases are based on much smaller conserved regions and do not require extensive similarity elsewhere in the sequence, as may be the case with whole-gene matches. More general characteristics of protein sequences, such as those of membrane proteins, can also be used to identify genes of interest. The rationale is that proteins involved in host interactions (likely to be virulence factors) should be localized to the cell surface or be secreted. Transmembrane sequences can be predicted by a variety of programs such as PHD (20,21); signal sequences can be identified with programs such as SIGNALP (22,23). Transmembrane and signal sequences and other characteristics are included in annotations in databases (e.g., the one for sexually transmitted disease pathogens) (Table 2).

Other sequence-based clues have been used in this type of analysis. Tandem repeats of simple (e.g., mono-, di-, tri-, or tetranucleotide) sequences are often found in or near certain virulence genes, called contingency genes (24,25). Because changes in the number of copies of repeats alter expression or other properties of these genes, leading to antigenic or other types of variation, this feature can be analyzed to identify genes. Finally, analysis of untranslated regulatory regions, though not extensive, appears to be a fruitful area for future studies. A genetic method for identifying new virulence factors is to find genes that are coregulated with known virulence factors (26). This type of analysis could be used in silico (analysis by computer). Motifs commonly associated with binding sites for regulators, such as inverted repeats, could be identified in regulatory regions of genes involved in pathogenesis or matching known virulence factors. These motifs could then be used to search for other regulatory regions containing the motif. The associated genes would then be candidates for virulence factors.

In summary, a number of strategies have been developed to mine genomic sequences for virulence factor genes. Other approaches will likely be developed. The availability of this information on easily accessible electronic databases will make this a routine tool in future studies of pathogenic microbes. All of these factors constitute a powerful set of new tools for research planning and experimental design and interpretation.

### Genetics Meets Genomics

One criticism of the sequence-gazing approach is that it is not hypothesis based. However, the theoretical analysis of genomic sequence described above requires laboratory validation of conclusions, which are the hypotheses that drive experimental design. The availability of sequence data not only generates hypotheses but also greatly speeds the task of testing them.

In systems with good genetics and suitable models to test virulence, the sequence allows design and construction of clones for making targeted knockout mutants—a type of mutation where a gene's function is knocked out by inserting DNA into or deleting the gene. These mutational methods are usually based on a polymerase chain reaction assay (PCR), since the sequence allows primers to be designed to amplify and clone the key sequences. In some organisms, wholesale construction of such mutants is under way (27). One can determine if inactivation of a gene leads to attenuation of infection in a model system. If genetic analysis is not feasible, it is still possible to test whether immunization with a gene product (either the whole protein or part of it) can lead to protection in a model. While this testing does not provide as strong a case for a role in virulence as a null mutant (a mutation that causes complete loss of function in a gene), it indicates whether the protein is a good vaccine target. In this case, the sequence allows design and construction of clones overexpressing the protein of interest in a more manipulable host (again by PCR amplification of key sequences). Often, identification and purification of proteins in the natural host are formidable tasks. However, whole-genome sequencing allows overproducers to be constructed in *Escherichia coli* or other workhorse strains.

Both of the methods described above can determine if a gene is functional when virulence is affected. However, when there is no effect, there is no indication of whether the gene is real or functional. Determining if the gene is transcribed and translated is then desirable. Reverse transcription (RT)-PCR, again basing primer design on the genome sequence, is often performed for such analysis and can be extended to determine operon structure in the genome. Genomewide transcription analysis is performed with DNA arrays. Protein prepared in a surrogate host can be used to detect antibodies in serum from infected persons, which is particularly relevant for surface protein candidates for immunodiagnostics. An immunopositive reaction indicates that a gene is transcribed and translated.

### Scanning for Function

The sequence-to-mutant method described above is appropriate when genes of interest can be identified by sequence analysis. However, there are likely to be novel genes that do not match known functions or domains and do not have characteristics used to identify surface proteins. How would one identify a secreted protein with a function not previously described and the sequence characteristics of a soluble protein? Or what about essential genes, targets for antimicrobial drugs, that may encode cytoplasmic proteins, some of which are novel and do not match known proteins? The methods described above would not be sufficient to identify these important functions.

Several methods that bridge this gap have been proposed for whole-genome function analysis. In all cases, the genome is scanned by exhaustive transposon mutagenesis, and mutants are screened en masse for functional properties. These methods can identify essential genes, virulence factors, and other types of phenotypes.

Genetic footprinting (28,29), which was developed for yeast, is also applicable to bacteria (Figure 1). This method depends on the complete genome sequence since PCR primers are made to the ends of each gene in the genome. A saturating set of transposon insertions is isolated at random in the genome, so all genes receive multiple insertions. The mutants are pooled, and the culture is split and grown under permissive and nonpermissive conditions. For essential genes, there is no permissive condition. For virulence functions, a permissive condition might be broth culture, and a nonpermissive condition might be an animal model. After growth, DNA is extracted from the cultures, and each mutant gene is assayed by PCR using one primer for the end of the gene and one primer for the end of the transposon. Each gene is assayed separately and generates a series of bands, each corresponding to a different insertion in the gene. Comparison of the permissive and nonpermissive conditions allows the identification of mutants that drop out (that is, do not grow) under nonpermissive conditions. An essential gene mutant gives no products in either permissive or nonpermissive samples. Mutants in a gene required for infection would give products with the permissive but not the nonpermissive culture. Other genes would give products under both conditions. In this way, one assays function by “knocking out” all genes.

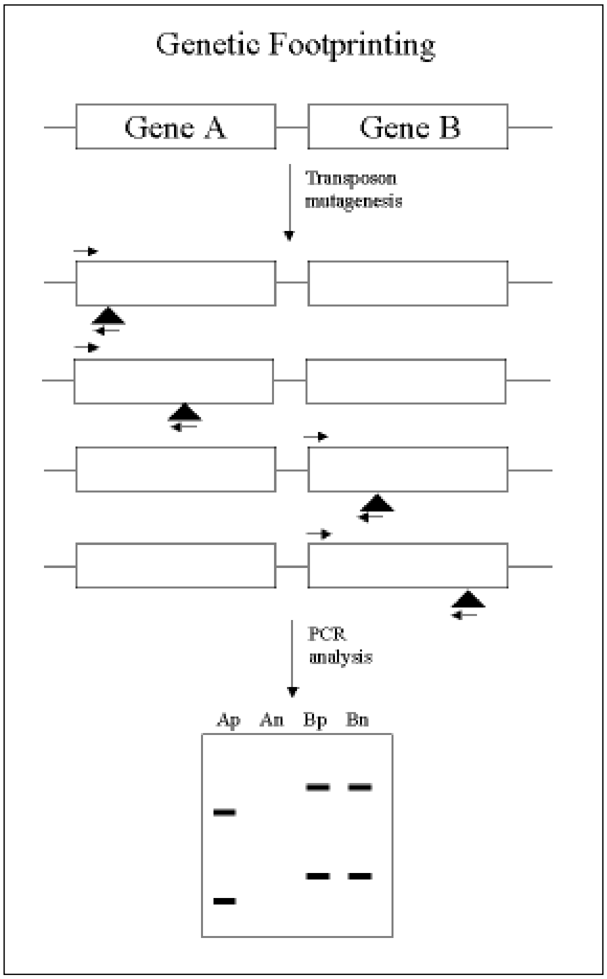

Figure 1. Genetic footprinting. Shown are two neighboring genes from the whole genome. Gene A encodes a virulence factor; gene B does not. Neither gene is essential. After transposon mutagenesis, multiple insertions (vertical triangles) are obtained in each gene (only two are shown). Polymerase chain reaction (PCR) primers (horizontal arrows) to the start of the gene and the transposon are used to amplify the sequences between insertion and start of gene. After electrophoresis, a characteristic set of bands is seen for each gene, corresponding to the location of insertions. Mutants of genes A and B grew under permissive conditions (Ap, Bp). Only mutants of gene B (Bn) grew under nonpermissive (animal model) conditions.

Signature-tagged mutagenesis (30) is another dropout mutant approach, but its scheme for tracking each gene differs (Figure 2). The transposon used for random mutagenesis has been prepared to have an index region in which each transposon has a different sequence. This region can be amplified by PCR. The resulting product can be used as a hybridization probe to uniquely identify the transposon that encodes it. The initial set of random insertion mutants is arrayed on a master and then pooled and grown

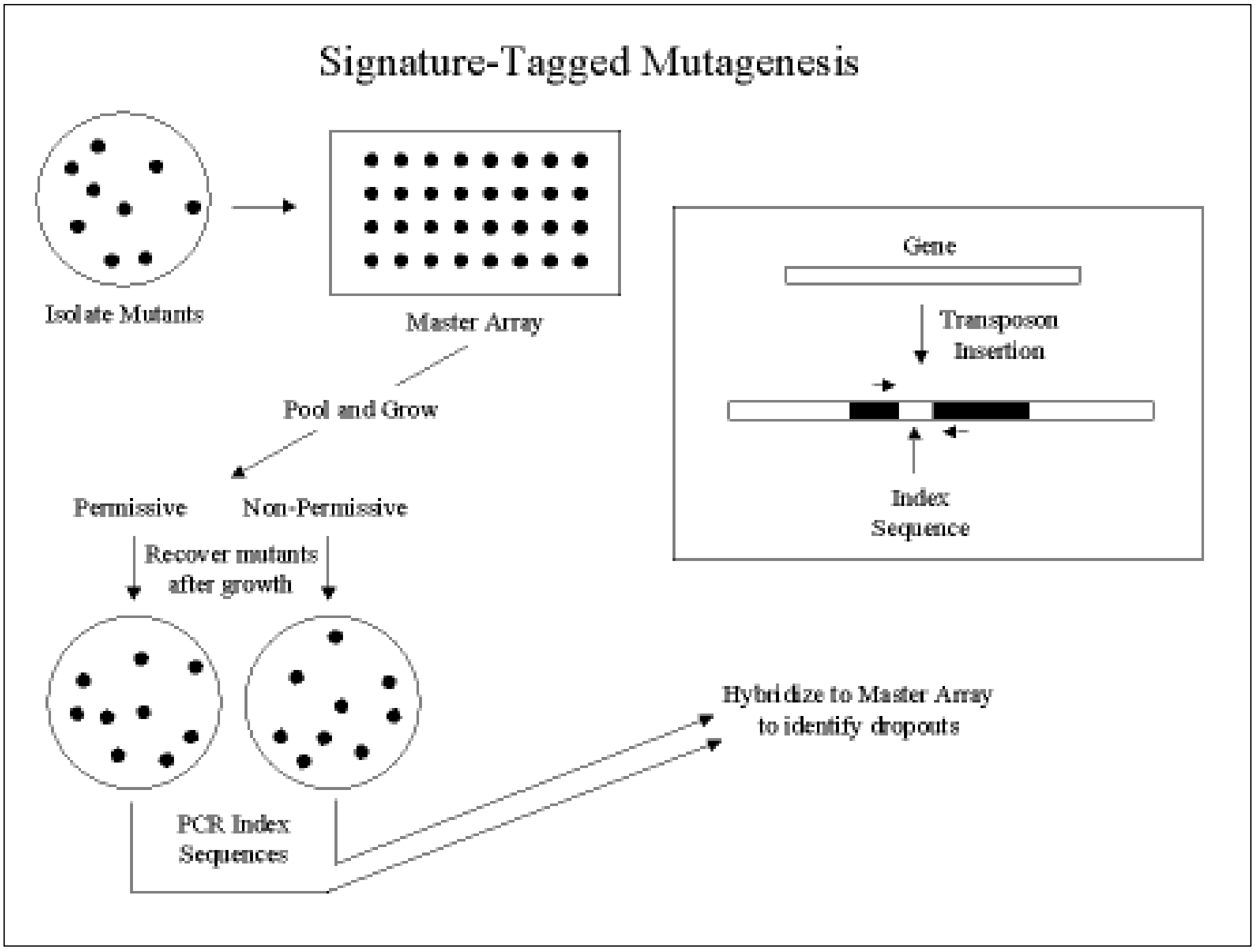

Figure 2. Signature-tagged mutagenesis. The box on the right shows a transposon insertion, indicating the index region and location of polymerase chain reaction (PCR) primers that amplify the segment unique to each transposon.

under permissive and nonpermissive conditions, as above. The mutants that emerge in each growth regimen are then collected, and their index regions are amplified and used to hybridize to the master array of original mutants. This process allows the identification of mutants that dropped out during the selection. Regions flanking the insertions in mutants of interest are then sequenced and compared to the genomic sequence to find inactivated gene(s). An important difference between signature-tagged mutogenesis and genetic footprinting is that in genetic footprinting each gene is specifically and systematically assayed, relying on the genome sequence. Thus, essential genes are readily found since they have no mutations. On the other hand, signature-tagged mutagenesis assays mutants randomly and thus could not determine that a gene could not be mutated until a large number of mutants had been tested. Nevertheless, this method has been widely used to detect virulence factor genes (31-36).

Additional methods using transposon scanning to find genes with essential or other functions will likely be developed. The methods described above often require more genetic manipulations than can be performed in some pathogenic organisms. Recent advances to overcome these limitations include using in vitro transposition to generate mutants (37) as well as new transposons with broad host ranges (38).

### One Genome Is Not Enough: Comparative Genomics

Comparative genomics, which requires input of multiple genomic sequences, is relatively new, and the microbial genome era is just entering truly large-scale production. The first whole-genome comparisons were of strains phylogenetically separated, since these were the only genomes available. Much can be learned about evolution from comparing such disparate organisms, but certain lessons can best be gleaned from comparing more closely related genomes. Recently, such comparisons have been performed with the genomes of *Mycoplasma genitalium* and *M. pneumoniae* (39,40), two strains of *Helicobacter pylori* (6), *Chlamydia trachomatis* and *C. pneumoniae* (2), and draft sequences of *Salmonella enterica* serotype Typhimurium (41) and *S.* Typhi (42) with the completed sequence of *E. coli*. These studies promise to provide pertinent, but different information about virulence functions than the analyses presented above. One type of comparison is between strains of the same genus that infect different tissues. This comparison results in lists of genes that are common or different; this outcome may ultimately be correlated with tissue-specific virulence factors. Moreover, genes that are common but not found in other genera may reflect unique morphologic characteristics as well as host interactions. A second type of comparison is between two strains of the same species. Here, one is identifying regions of variability that are to be avoided in choosing targets for vaccine or antimicrobial therapy and that may be less important in infection. This is one of the newer and very promising areas in microbial genomics. Web sites that provide genomic data will also likely provide methods of comparative analyses, similar to methods provided by the Bugspray feature on the sexually transmitted diseases database site.

### Solutions without Answers

If the ultimate aim of pathogen genome sequencing is the development of vaccines, therapeutics, and diagnostics, candidate genes may be identified before the mechanism of infection is understood. The genome sequence is the “parts list,” used to test each gene product for its potential usefulness by various high-throughput methods. DNA vaccines constitute one of the few documented approaches for this purpose (43-45). In this case, genes targeted for vaccine use are cloned in expression vectors, and their efficacy for vaccine use is tested without ever studying the gene product. The potential of this approach was shown with Mycoplasma. A more commonly tried method in industry, often presented at conferences although not published, is to express a subset of the total set of genes in *E. coli*, purify the products, and test them in a mouse or other small animal model. The subset of genes is usually selected by computational criteria, i.e., their similarity to known virulence genes or indications that the protein is surface localized or secreted. In addition, expression analysis, using array technology, for instance, is often used to identify genes expressed in the host. Furthermore, many organism-specific genes without database matches are included in the subset, which may comprise 500 to 1,000 genes. Expression in *E. coli* is accomplished by using standard vectors, but usually as a fusion protein to a component that can simplify purification (histidine-tag, glutathione-S-transferase, or thioredoxin, for example). Many genes may fall by the wayside because of difficulties in expression or purification, but even if only 10% make it through, at least 50 to 100 candidates are available for testing in animal models. Such a large number of candidates easily surpasses the number of proteins identified for testing by traditional means. Clearly, discovering genes to test no longer limits the identification of useful gene products; rather, the new bottleneck is finding suitable models for high-throughput testing of efficacy. In any event, it is likely that candidate genes will be identified and enter industrial development long before researchers understand their role in infection.

### Acknowledgments

The author thanks Steven Norris, Claire Fraser, and Richard Gibbs for excellent collaboration on several genome projects; Erica Sodergren and Tim Palzkill for many useful discussions; and the National Institutes of Health for support.

Dr. Weinstock is professor of microbiology and molecular genetics and codirector of the Center for the Study of Emerging and Re-emerging Pathogens at the University of Texas, Houston Medical School. He is also codirector of the Human Genome Sequencing Center, Baylor College of Medicine, Houston, Texas. His research interests include applications of genetics and genomics to problems in microbiology, high-throughput DNA sequencing of the human, mouse, and other large genomes, and bioinformatics.

### References

1. Fraser CM, Casjens S, Huang WM, Sutton GG, Clayton R, Lathigra R, et al. Genomic sequence of a Lyme disease spirochaete, *Borrelia burgdorferi*. Nature 1997;390:580-6.

2. Kalman S, Mitchell W, Marathe R, Lammel C, Fan J, Hyman RW, et al. Comparative genomes of *Chlamydia pneumoniae* and *C. trachomatis*. Nat Genet 1999;21:385-9.
3. Stephens RS, Kalman S, Lammel C, Fan J, Marathe R, Aravind L, et al. Genome sequence of an obligate intracellular pathogen of humans: *Chlamydia trachomatis*. Science 1998;282:754-9.
4. Blattner FR, Plunkett G III, Bloch CA, Perna NT, Burland V, Riley M, et al. The complete genome sequence of *Escherichia coli* K-12. Science 1997;277:1453-74.
5. Fleischmann RD, Adams MD, White O, Clayton RA, Kirkness EF, Kerlavage AR, et al. Whole-genome random sequencing and assembly of *Haemophilus influenzae* Rd [see comments]. Science 1995;269:496-512.
6. Alm RA, Ling LS, Moir DT, King BL, Brown ED, Doig PC, et al. Genomic-sequence comparison of two unrelated isolates of the human gastric pathogen *Helicobacter pylori* [published erratum appears in Nature 1999 Feb 25;397:719]. Nature 1999;397:176-80.
7. Tomb JF, White O, Kerlavage AR, Clayton RA, Sutton GG, Fleischmann RD, et al. The complete genome sequence of the gastric pathogen *Helicobacter pylori* [published erratum appears in Nature 1997 Sep 25;389:412]. Nature 1997;388:539-47.
8. Cole ST, Brosch R, Parkhill J, Garnier T, Churcher C, Harris D, et al. Deciphering the biology of *Mycobacterium tuberculosis* from the complete genome sequence [published erratum appears in Nature 1998 Nov 12;396:190]. Nature 1998;393:537-44.
9. Fraser CM, Gocayne JD, White O, Adams MD, Clayton RA, Fleischmann RD, et al. The minimal gene complement of *Mycoplasma genitalium*. Science 1995;270:397-403.
10. Himmelreich R, Hilbert H, Plagens H, Pirkl E, Li BC, Herrmann R. Complete sequence analysis of the genome of the bacterium *Mycoplasma pneumoniae*. Nucleic Acids Res 1996;24:4420-49.
11. Andersson SG, Zomorodipour A, Andersson JO, Sicheritz-Ponten T, Alsmark UC, Podowski RM, et al. The genome sequence of *Rickettsia prowazekii* and the origin of mitochondria. Nature 1998;396:133-40.
12. Fraser CM, Norris SJ, Weinstock GM, White O, Sutton GG, Dodson R, et al. Complete genome sequence of *Treponema pallidum*, the syphilis spirochete. Science 1998;281:375-88.
13. Altschul SF, Madden TL, Schaffer AA, Zhang J, Zhang Z, Miller W, et al. Gapped BLAST and PSI-BLAST: a new generation of protein database search programs. Nucleic Acids Res 1997;25:3389-402.
14. Altschul SF, Gish W, Miller W, Myers EW, Lipman DJ. Basic local alignment search tool. J Mol Biol 1990;215:403-10.
15. Weinstock GM, Hardham JM, McLeod MP, Sodergren EJ, Norris SJ. The genome of *Treponema pallidum*: new light on the agent of syphilis. FEMS Microbiol Rev 1998;22:323-32.
16. Henikoff JG, Henikoff S, Pietrokovski S. New features of the Blocks Database servers. Nucleic Acids Res 1999;27:226-8.
17. Henikoff S, Henikoff JG. Protein family classification based on searching a database of blocks. Genomics 1994;19:97-107.
18. Corpet F, Gouzy J, Kahn D. The ProDom database of protein domain families. Nucleic Acids Res 1998;26:323-6.
19. Corpet F, Gouzy J, Kahn D. Recent improvements of the ProDom database of protein domain families. Nucleic Acids Res 1999;27:263-7.
20. Rost B, Fariselli P, Casadio R. Topology prediction for helical transmembrane proteins at 86% accuracy. Protein Sci 1996;5:1704-18.
21. Rost B. PHD: predicting one-dimensional protein structure by profile-based neural networks. Methods Enzymol 1996;266:525-39.
22. Claros MG, Brunak S, von Heijne G. Prediction of N-terminal protein sorting signals. Curr Opin Struct Biol 1997;7:394-8.
23. Nielsen H, Engelbrecht J, Brunak S, von Heijne G. Identification of prokaryotic and eukaryotic signal peptides and prediction of their cleavage sites. Protein Eng 1997;10:1-6.
24. Saunders NJ, Peden JF, Hood DW, Moxon ER. Simple sequence repeats in the *Helicobacter pylori* genome. Mol Microbiol 1998;27:1091-8.
25. Hood DW, Deadman ME, Jennings MP, Bisercic M, Fleischmann RD, Venter JC, et al. DNA repeats identify novel virulence genes in *Haemophilus influenzae*. Proc Natl Acad Sci U S A 1996;93:11121-5.
26. Taylor RK, Miller VL, Furlong DB, Mekalanos JJ. Use of phoA gene fusions to identify a pilus colonization factor coordinately regulated with cholera toxin. Proc Natl Acad Sci U S A 1987;84:2833-7.
27. Link AJ, Phillips D, Church GM. Methods for generating precise deletions and insertions in the genome of wild-type *Escherichia coli*: application to open reading frame characterization. J Bacteriol 1997;179:6228-37.
28. Smith V, Chou KN, Lashkari D, Botstein D, Brown PO. Functional analysis of the genes of yeast chromosome V by genetic footprinting. Science 1996;274:2069-74.
29. Smith V, Botstein D, Brown PO. Genetic footprinting: a genomic strategy for determining a gene's function given its sequence. Proc Natl Acad Sci U S A 1995;92:6479-83.
30. Hensel M, Shea JE, Gleeson C, Jones MD, Dalton E, Holden DW. Simultaneous identification of bacterial virulence genes by negative selection. Science 1995;269:400-3.
31. Edelstein PH, Edelstein MA, Higa F, Falkow S. Discovery of virulence genes of *Legionella pneumophila* by using signature tagged mutagenesis in a guinea pig pneumonia model. Proc Natl Acad Sci U S A 1999;96:8190-5.
32. Darwin AJ, Miller VL. Identification of *Yersinia enterocolitica* genes affecting survival in an animal host using signature-tagged transposon mutagenesis. Mol Microbiol 1999;32:51-62.
33. Hensel M. Whole genome scan for habitat-specific genes by signature-tagged mutagenesis. Electrophoresis 1998;19:608-12.
34. Chiang SL, Mekalanos JJ. Use of signature-tagged transposon mutagenesis to identify *Vibrio cholerae* genes critical for colonization. Mol Microbiol 1998;27:797-805.

35. Mei JM, Nourbakhsh F, Ford CW, Holden DW. Identification of *Staphylococcus aureus* virulence genes in a murine model of bacteraemia using signature-tagged mutagenesis. Mol Microbiol 1997;26:399-407.
36. Lehoux DE, Sanschagrin F, Levesque RC. Defined oligonucleotide tag pools and PCR screening in signature-tagged mutagenesis of essential genes from bacteria. Biotechniques 1999;26:473-8, 480.
37. Akerley BJ, Rubin EJ, Camilli A, Lampe DJ, Robertson HM, Mekalanos JJ. Systematic identification of essential genes by in vitro mariner mutagenesis. Proc Natl Acad Sci U S A 1998;95:8927-32.
38. Rubin EJ, Akerley BJ, Novik VN, Lampe DJ, Husson RN, Mekalanos JJ. In vivo transposition of mariner-based elements in enteric bacteria and mycobacteria. Proc Natl Acad Sci U S A 1999;96:1645-50.
39. Herrmann R, Reiner B. *Mycoplasma pneumoniae* and *Mycoplasma genitalium*: a comparison of two closely related bacterial species. Curr Opin Microbiol 1998;1:572-9.
40. Himmelreich R, Plagens H, Hilbert H, Reiner B, Herrmann R. Comparative analysis of the genomes of the bacteria *Mycoplasma pneumoniae* and *Mycoplasma genitalium*. Nucleic Acids Res 1997;25:701-12.
41. Wong RM, Wong KK, Benson NR, McClelland M. Sample sequencing of a *Salmonella typhimurium* LT2 lambda library: comparison to the *Escherichia coli* K12 genome. FEMS Microbiol Lett 1999;173:411-23.
42. McClelland M, Wilson RK. Comparison of sample sequences of the *Salmonella* typhi genome to the sequence of the complete *Escherichia coli* K-12 genome. Infect Immun 1998;66:4305-12.
43. Barry MA, Lai WC, Johnston SA. Protection against mycoplasma infection using expression-library immunization. Nature 1995;377:632-5.
44. Lai WC, Bennett M, Johnston SA, Barry MA, Pakes SP. Protection against *Mycoplasma pulmonis* infection by genetic vaccination. DNA Cell Biol 1995;14:643-51.
45. Johnston SA, Barry MA. Genetic to genomic vaccination. Vaccine 1997;15:808-9.
